# Conceptual metaphorical mapping in chimpanzees (*Pan troglodytes*)

**DOI:** 10.7554/eLife.00932

**Published:** 2013-10-22

**Authors:** Christoph D Dahl, Ikuma Adachi

**Affiliations:** 1 Section of Language and Intelligence, Primate Research Institute, Kyoto University, Inuyama, Japan; 2 Department of Psychology, National Taiwan University, Taipei, Taiwan; 3 Center for International Collaboration and Advanced Studies in Primatology, Primate Research Institute, Kyoto University, Inuyama, Japan; Oxford University, United Kingdom

**Keywords:** chimpanzee, conceptual metaphorical mapping, cross-modal mapping, language, linguistic, hierarchy, Other

## Abstract

Conceptual metaphors are linguistic constructions. Such a metaphor is humans’ mental representation of social rank as a pyramidal-like structure. High-ranked individuals are represented in higher positions than low-ranked individuals. We show that conceptual metaphorical mapping between social rank and the representational domain exists in our closest evolutionary relatives, the chimpanzees. Chimpanzee participants were requested to discriminate face identities in a vertical arrangement. We found a modulation of response latencies by the rank of the presented individual and the position on the display: a high-ranked individual presented in the higher and a low-ranked individual in the lower position led to quicker identity discrimination than a high-ranked individual in the lower and a low-ranked individual in the higher position. Such a spatial representation of dominance hierarchy in chimpanzees suggests that a natural tendency to systematically map an abstract dimension exists in the common ancestor of humans and chimpanzees.

**DOI:** http://dx.doi.org/10.7554/eLife.00932.001

## eLife digest

It is thought that the ability to connect an abstract concept to something physical helps us to understand abstract ideas more easily. Examples include the use of conceptual metaphors that draw parallels between something abstract, such as social status, and physical position, even though there is no connection between them: familiar examples include phrases such as ‘top dog’ or ‘upper class’. It has long been assumed that the use of such conceptual metaphors is uniquely human.

Many social animals have hierarchies of dominance within groups, with particular individuals being ranked above or below other individuals. Chimpanzees—our closest relatives in the animal kingdom—are a good example of this, and although their cognitive processes are known to be similar to those of humans in many ways, we do not know if they make use of conceptual metaphors. Moreover, we don’t even know if conceptual metaphors can exist in the absence of language.

When researchers want to investigate how concepts are cognitively linked in the brain, they often use ‘coherent’ or ‘incoherent’ stimuli. A good example of an incoherent stimulus would be the word ‘red’ printed in blue ink. Because our neural representations of the colour blue and the word blue are linked, it is harder for a person to read the word red when it is printed in blue than when it is printed in red (which would be a coherent stimulus).

To test whether chimpanzees use a conceptual metaphor in which social status corresponds to height, Dahl and Adachi showed six chimpanzees photographs of four other chimpanzees who were known to them, and tested whether the relative positions of the photographs affected the ability of the chimpanzees to identify which of the two photographs they had been shown earlier. For example, a photograph of a high-ranked, dominant chimpanzee could be shown above a photograph of a lower-ranked chimpanzee (a coherent stimulus) or below a photograph of a lower-ranked chimpanzee (an incoherent stimulus). The chimpanzees doing the tests had to identify which of the photographs they had been shown earlier by touching the correct photograph on a screen.

Dahl and Adachi found that it took longer for chimpanzees to complete the task when the photograph was in the ‘wrong’ position. This suggests that the neural representations of social status and physical position might be linked in chimpanzees. If the social status and the physical position of the photograph match, the chimpanzee doing the test can quickly identify the photograph that it has been shown earlier. However, if they do not match, the conflict between the neural representations of social status and physical position slows down the response. These findings suggest that conceptual metaphors are not uniquely human and, moreover, that they could have emerged before the development of language.

**DOI:** http://dx.doi.org/10.7554/eLife.00932.002

## Introduction

“high” vs “low status”, “top of the heap”, “bottom of the barrel”: These or similar expressions are widely observed across cultures and languages (Pinker, 1997). The cross-modal correspondence between the visuospatial domain (high or low) and an abstract domain (rank) has been described as a conceptual metaphor (Lakoff and Johnson, 1980a, b) and thought to be uniquely human (Feldman and Narayanan, 2004). A conceptual metaphor takes one concept and connects that to another concept in order to better understand that concept. The way we think and act is largely influenced by conceptual metaphors, even without being fully aware of them (Lakoff and Johnson, 1980a). The question remains if conceptual metaphorical mapping is indeed uniquely human or if it appears in other primates and thus describes a conceptual metaphorical mapping that predates language. To answer this question, we examined if our evolutionary closest relatives, the chimpanzees, have conceptual metaphors as we humans do.

Decades of research have shown that neural representations of objects and entities exist in monkeys (Sigala et al., 2002), apes (Fukushima et al., 2010) and humans (Kourtzi and Kanwisher, 2001). The abilities of object representation and recognition further apply to social domains such as recognition of faces (Dahl et al., 2007; Dahl et al., 2013), conspecifics (Dahl et al., 2007), and ingroup-outgroup members (Pokorny and de Waal, 2009). To avoid or reduce costly social conflicts among individuals, a crucial skill for living in social groups is to express one’s status and to recognize the status of the others via visual or vocal cues (Paxton et al., 2010). Recognition of status or rank allows inferences about expected roles of oneself and of others during group situations (Ridgeway and Diekema, 1989). Access to food resources, information and social respect are facilitated with high rank in the hierarchy, while some degree of protection and care are granted to lower-ranked individuals (Fiske, 1992). In many non-human species, spatial information, such as perceived physical body size, and facial and body postures, serve as an indicator of social status (Maestripieri, 1996; Tiedens and Fragale, 2003). Besides these non-verbal cues, we humans developed a conceptual metaphor, which connects social rank to the spatial domain (e.g., “high” vs “low status” [Pinker, 1997]). Accordingly, humans represent social status in a pyramidal-like structure (Bosserman, 1983): high-ranked individuals are represented in spatially higher positions than low-ranked individuals in diverse contexts, for example, stratification (Saunders, 1989), organizations (Weber, 1991), religion, family, human needs (Maslow, 1943), and others. In this study, we examined if such conceptual metaphorical mapping between social dominance and the spatial domain is uniquely human or if it appears in our evolutionary closest relative, the chimpanzees. We addressed this question by comparing response latencies on discriminating photographs of familiar chimpanzee faces of high- and low-ranked individuals in a vertically aligned delayed matching-to-sample task. We hypothesize that *coherent* arrangements, such as a high-ranked individual presented in the higher and a low-ranked individual presented in the lower position, lead to quicker identity discrimination than *incoherent* arrangements, such as a high-ranked individual in the lower and a low-ranked individual in the higher position.

## Results

**Figure 1.**
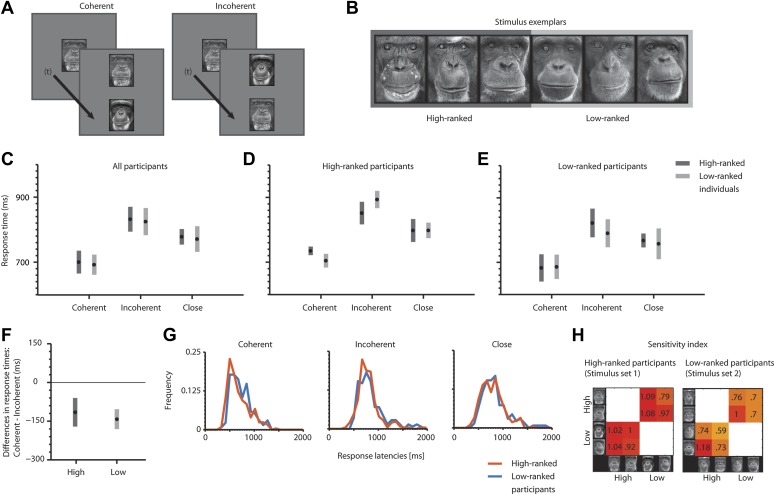
Task sequence, example stimuli and response latency analyses. (**A**) Typical trial sequence. (**B**) Stimulus exemplars. (**C**) Average response latencies for *coherent*, *incoherent* and *close* trials (mean ± SEM) for all participants, (**D**) for high-ranked participants and (**E**) for low-ranked participants. (**F**) Average response latency differences (*coherent*–*incoherent*) for *high* and *low* positions. (**C**, **F**) The number of independent data points (N) is six for each condition. (**G**) Normalized frequency distribution of response latencies of high- and low-ranked participants for *coherent*, *incoherent* and *close* trials. (**H**) Sensitivity index for both stimulus sets and stimuli. Positive values indicate facilitation for *coherent* relative to *incoherent* trials. **DOI:** http://dx.doi.org/10.7554/eLife.00932.003

We sequentially presented a cue (individual 1, 750 ms) followed by an inter-stimulus interval (500 ms) and two vertically arranged stimuli (match: individual 1; distractor: individual 2) (Figure 1A,B). The chimpanzees were required to indicate which of the two simultaneously presented faces (match, distractor) corresponds to the initially presented face (cue) in identity by touching one of the faces. Note that the participants did *not* classify the stimuli based on social rank. In addition to *coherent* and *incoherent* combinations of *stimulus* and *position*, we included a neutral condition combining two pictures of closely ranked individuals in a trial (*close* condition). In the first step of analyses, we pooled response latencies for *coherence* (*coherent* vs *incoherent* vs *close*) and *position* (*high* vs *low*). Using a mixed model ANOVA with *coherence* and *position* as fixed factors, we found a main effect for *coherence* (*F*(2, 30) = 5, m.s.e. = 1.10e+004, p<0.001), but not for *position* (p>0.34) or the interaction between the two factors (p>0.83) (Figure 1C). Post-hoc t-tests (Bonferroni-corrected for multiple comparisons) revealed a significant response facilitation for *coherent* as opposed to *incoherent* trials (*t*(10) = -3.20, m.s.e. = 8.60e+003, p<0.01 [one-tailed]) and *close* trials (*t*(10) = −1.92, m.s.e. = 1.14e+004, p<0.05 [one-tailed]). However, there was no significant deterioration for *incoherent* compared to *close* trials (p>0.11) (Figure 1C). In the second step of analyses, we pooled response latencies for *position* (*high* vs *low*) and compared the differences of *coherent* and *incoherent* trials in one-sample *t*-tests. We found significant deviations from zero for the *high* position (*t*(5) = 16.64, m.s.e. = 1.11e+004, p<0.001 [one-tailed]) and for the low position (t(5) = 20.13, m.s.e. = 6.88e+003, p<0.001 [one-tailed]) (Figure 1F). Further, there was no significant difference between the positions *high* and *low* (p>0.55).

We further split the participants into two groups according to their own ranks. This is equivalent to a separation by the stimulus sets (set 1 for the high-ranked and set 2 for the low-ranked participants, see ‘Matarials and methods’). High-ranked participants showed the same response patterns as low-ranked participants (Figure 1D,E). Due to the low sample size, we were unable to run statistical tests across participants; however, we collapsed all data samples of high-ranked participants for *coherent*, *incoherent* and *close* conditions and compared them to the corresponding conditions in low-ranked participants (Figure 1G). Two-sample Kolmogorov-Smirnov tests revealed that none of the frequency distributions of response latencies were statistically different across the two participant groups (all p>0.54). In addition, we calculated a sensitivity index reflecting the amount to which two sample distributions are separable from each other (also referred to as d-prime). We again split the data according to the rank of the individuals (high- vs low-ranked) (equivalent to splitting according to the stimulus sets). We further binned data samples (response latencies) across participants for *coherent* and *incoherent* trials. We then calculated the sensitivity index for each stimulus in combination with each other stimulus with which it occurred in the task (Figure 1H, x-axis = *distractor*; y-axis = *cue* and *match*) by considering the means and standard deviations of the corresponding data bins of *coherent* and *incoherent* trials (Equation 1, ‘Materials and methods’). Positive values illustrate response facilitation for coherent trials relative to incoherent trials. As indicated in Figure 1D,E, there is more variance in the responses of the low-ranked (Figure 1E, four participants) than the high-ranked participants (Figure 1D, two participants), leading to a lower sensitivity in low-ranked participants (*t*(14) = 2.53, m.s.e. = 8.36e+003, p<0.05, Figure 1H). Importantly, all stimuli elicited sensitivity scores in accordance with the hypothesis, that is, coherent stimulus arrangements led to response facilitation and incoherent stimulus arrangements led to response deterioration, indicated by positive values.

## Discussion

We showed that in chimpanzees discrimination performances between familiar conspecific faces are systematically modulated by the location and the social status of the presented individuals, leading to discrimination facilitation or deterioration. *Coherent* arrangements as opposed to *incoherent* arrangements led to a facilitation of recognition. Further, both, a high-ranked individual at the higher position and a low-ranked individual at the lower position caused recognition facilitation equivalently. Importantly, the participants were not trained on discriminating the ranks of the presented individuals. Instead, they were substantially affected by the rank while discriminating the identity of those individuals. The modulations are in accordance with a spatial arrangement representing high-ranked individuals at the top and low-ranked individuals at the bottom, hence reflecting the inverse function of response latencies and spatial distance of two individuals on the display and in the mental hierarchy space of the participants.

It has to be noted that a confound between perceptual and conceptual determinants might exist. We accounted for potential confounding perceptual cues, such as physical height, gestures and postures (Ridgeway and Diekema, 1989; Paxton et al., 2010). However, teasing apart perceptual and conceptual determinants entirely is almost impossible. There might be a co-dependence of the two domains, with the conceptual domain having been established on the basis of the perceptual domain. We applied the following control for a perceptual confound: If perceptual cues cause the effect, for example high-ranked individuals would naturally appear in higher positions than low-ranked individuals, the response characteristics of high-ranked and low-ranked participants would be different due to the rank difference between participant and the presented individuals: A high-ranked participant is more likely to show response facilitation for low-ranked individuals, while a low-ranked participant is more likely to show response facilitation for high-ranked individuals. This, however, is not the case. Response characteristics are similar in high-ranked and low-ranked participants. Thus, even though perceptual determinants cannot be fully excluded with this control, it is still suggestive that the effect is not merely based on perceptual cues but on, to some extent, conceptual components.

In addition, we controlled for a differential effect due to one of the two stimulus sets used in the experiment. We showed that individual stimuli elicited a comparable effect within and across stimulus sets by estimating sensitivity indices. In other words, the two stimulus sets contributed equally to the effect.

A spatial component of representation has been shown in other domains: for example in humans’ responses to low-digit numbers are faster with a left-side button-press whereas higher digits are categorized faster when right-side button-presses are required (Dehaene et al., 1993). Moreover, merely looking at number causes a shift of attention to the left or the right side (Fischer et al, 2003). In other words, the mental number line reflects a cross-modal mapping of visual cues (numbers) and cognitive labels (values). Interestingly, social status and number comparisons recruit to some extent overlapping neural substrates in the intraparietal sulci of the human brain (Chiao et al., 2009). There is evidence for a non-verbal, supramodal neural representation of numerosity in the macaque ventral intraparietal sulcus and lateral prefrontal cortex (Nieder, 2012); however, neural evidence for cross-modal correspondences is missing. Rare evidence for non-human cross-modal correspondences comes from visuo-auditory mappings between high luminance and high pitch in chimpanzees (Ludwig et al., 2011). This relationship between luminance and pitch illustrates a form of sound-symbolism, which refers to the concept that in human language words and referents are not arbitrary (Nuckolls, 1999). Hence, while the existence of such a systematic mapping between luminance and pitch in chimpanzee suggests the emergence of an early vocabulary of human language (Ramachandran and Hubbard, 2001), we here expand this finding to a cross-modal correspondence between vision and an abstract domain: the social status. A natural tendency to systematically map an abstract dimension, such as social status, in our closest and nonlinguistic relatives, the chimpanzees, suggests that this tendency had already evolved in the common ancestors of humans and chimpanzees. This tendency might have influenced the emergence of metaphorical linguistics, thinking via image schema, an embodied pre-linguistic structure of experience that motivates conceptual metaphor mappings (Lakoff and Johnson, 1980a). According to Lakoff (Lakoff and Johnson, 1980b), orientational metaphors, such as ‘more is up’, ‘good is up’ and ‘dominant is up’, are based on observational correlation between increasing a substance and seeing the level of the substance rise, like adding an element to a pile. Given the strong physical basis, these metaphors are good candidate for universal concepts. Until now, conceptual metaphors have been exclusive human experiences. Our findings point in a different direction.

## Materials and methods

Six chimpanzees (*Pan troglodytes*; 1 male juvenile, 2 female juveniles [both around 11 years] and 3 female adults [both around 31 years]) participated in this study. The chimpanzees live in groups of 14 individuals with access to environmentally enriched outdoor (770 m^2^) as well as indoor compounds. The chimpanzees participated in a variety of computer-controlled tasks in the past (Matsuzawa, 2003; Matsuzawa et al., 2006). They are experienced in horizontally aligned delayed matching-to-sample (DMS) tasks; however, they are inexperienced in a vertical version of a DMS task. Effects of training in the vertically aligned DMS task can be ruled out.

The vertical spacing of the match and distractor stimuli was about 70 mm. Stimuli were presented at a 17-inch LCD touch panel display (1280 × 1024 pixels) controlled by custom-written software under Visual Basic 2010 (Microsoft Corporation, Redmond, Washington, USA). The stimulus size was approximately 4.5 by 6° of gaze angel. One degree of gaze angle corresponded to approximately 0.86 cm on the screen at 50 cm viewing distance. Below the display a food tray was installed in which pieces of food reward were delivered by a custom-designed feeder after completion of a correct trial. Chimpanzee participants sat in an experimental booth (2.5 m wide, 2.5 m deep, 2.1 m high), with the experimenter and the participants separated by transparent acrylic panels.

We used photographs of faces of chimpanzee individuals with obvious dominant or submissive social ranks. The face pictures were taken from individuals familiar to the participants. All faces were normalized for luminance and contrast. The agreement of twenty independent raters (researchers and caretakers familiar with the chimpanzees at the Primate Research Institute) on the social ranks of the chimpanzees was found to be Kappa = 1.00 (p<0.001), 95% CI (1.00, 1.00). For the six participants (*Pan troglodytes*; 1 male adolescent, 2 female adolescents and 3 female adults), the face stimuli varied according to the group particular participants belonged to. We only presented face stimuli from the same group as the participant. In total, we used two sets of four face stimuli each: stimulus set 1 for two participants and stimulus set 2 for four participants. Under the assumptions that high-rank individuals presented in spatially higher position and low-ranked individuals presented in spatially lower position lead to *faster* identification, while high-rank individuals in lower position and low-ranked individual in higher position lead to *slower* identification, we designed the experiment according to the conditions *coherent* and *incoherent*, referring to the coherence between social rank and spatial position of presentation on the screen. In addition, we compared stimuli of close distance in social rank, here referred to as the *close* condition, serving as the baseline condition. For this condition, there is no prediction for a crossmodal association of social rank and spatial position. The order of experimental conditions and the spatial position of match and distractor stimuli were counterbalanced. Each participant did six blocks with 48 trials each. Only correct trials went into the analyses, which is 69% (±4.5% S.E.M.) of all trials.

The dependent variable was response latencies. We conducted an analysis of variances among the participants using a mixed model ANOVA, with *coherence* (coherent, incoherent and close conditions) and *position* as a fixed factors (high, low) as well as two-sample t-test (Bonferroni-corrected for multiple comparisons) to compare individual experimental conditions. For the post-hoc analysis of *coherence*, we collapsed the data samples from high and low positions for all three conditions (*coherent*, *incoherent* and *close*) of each participant (N = 6). For the post-hoc analysis of *position*, we subtracted the incoherent condition from the coherent condition for *high* and *low* positions of each participant (N = 6). Further, the distribution of response latencies for each condition was binned into 12 equally sized bins and normalized by dividing the absolute frequency (i.e., the number of events in each bin) by the total number of occurrences, resulting in the relative frequency with values ranging from 0 to 1. These normalized distributions were compared using two-sample Kolmogorov-Smirnov tests. To compare the outcome by the two stimulus sets, we did the following analytical procedure: We split the data samples according to the stimulus set used for the participants and according the conditions *coherent* and *incoherent*. These four data sets were then binned according to all combinations of stimuli as they occurred in the experiment. In other words, we binned the response latencies for each stimulus showing a high-ranked individual in combination with both stimuli showing low-ranked individuals and, visa-versa, for each stimulus showing a low-ranked individual in combination with both stimuli showing high-ranked individuals (Figure 1H). For both stimulus sets we took the distributions of response latencies for each stimulus combination of *coherent* and *incoherent* conditions and determined a sensitivity index, describing the separation of these distributions under consideration of the standard deviation using the following equation:

(1) $$\text{idx}\;=\;\frac{\mu_{c1}-\;\mu_{c2}}{\sqrt{\frac{1}{2}\left(\sigma_{c1}^{2}\;+\;\sigma_{c2}^{2}\;\right)}}$$

with *c1* and *c2* being two experimental conditions, *µ* the mean and *σ* the standard deviation. Positive values indicate facilitation for *coherent* above *incoherent* trials.
